# Synergy Between Public and Private Health Care Organizations During COVID-19 on Twitter: Sentiment and Engagement Analysis Using Forecasting Models

**DOI:** 10.2196/37829

**Published:** 2022-08-18

**Authors:** Aditya Singhal, Manmeet Kaur Baxi, Vijay Mago

**Affiliations:** 1 Department of Computer Science Lakehead University Thunder Bay, ON Canada

**Keywords:** social media, health care, Twitter, content analysis, user engagement, sentiment forecasting, natural language processing, public health, pharmaceutical, public engagement

## Abstract

**Background:**

Social media platforms (SMPs) are frequently used by various pharmaceutical companies, public health agencies, and nongovernment organizations (NGOs) for communicating health concerns, new advancements, and potential outbreaks. Although the benefits of using them as a tool have been extensively discussed, the online activity of various health care organizations on SMPs during COVID-19 in terms of engagement and sentiment forecasting has not been thoroughly investigated.

**Objective:**

The purpose of this research is to analyze the nature of information shared on Twitter, understand the public engagement generated on it, and forecast the sentiment score for various organizations.

**Methods:**

Data were collected from the Twitter handles of 5 pharmaceutical companies, 10 US and Canadian public health agencies, and the World Health Organization (WHO) from January 1, 2017, to December 31, 2021. A total of 181,469 tweets were divided into 2 phases for the analysis, before COVID-19 and during COVID-19, based on the confirmation of the first COVID-19 community transmission case in North America on February 26, 2020. We conducted content analysis to generate health-related topics using natural language processing (NLP)-based topic-modeling techniques, analyzed public engagement on Twitter, and performed sentiment forecasting using 16 univariate moving-average and machine learning (ML) models to understand the correlation between public opinion and tweet contents.

**Results:**

We utilized the topics modeled from the tweets authored by the health care organizations chosen for our analysis using nonnegative matrix factorization (NMF): c_umass_=–3.6530 and –3.7944 before and during COVID-19, respectively. The topics were chronic diseases, health research, community health care, medical trials, COVID-19, vaccination, nutrition and well-being, and mental health. In terms of user impact, WHO (user impact=4171.24) had the highest impact overall, followed by public health agencies, the Centers for Disease Control and Prevention (CDC; user impact=2895.87), and the National Institutes of Health (NIH; user impact=891.06). Among pharmaceutical companies, Pfizer’s user impact was the highest at 97.79. Furthermore, for sentiment forecasting, autoregressive integrated moving average (ARIMA) and seasonal autoregressive integrated moving average with exogenous factors (SARIMAX) models performed best on the majority of the subsets of data (divided as per the health care organization and period), with the mean absolute error (MAE) between 0.027 and 0.084, the mean square error (MSE) between 0.001 and 0.011, and the root-mean-square error (RMSE) between 0.031 and 0.105.

**Conclusions:**

Our findings indicate that people engage more on topics such as COVID-19 than medical trials and customer experience. In addition, there are notable differences in the user engagement levels across organizations. Global organizations, such as WHO, show wide variations in engagement levels over time. The sentiment forecasting method discussed presents a way for organizations to structure their future content to ensure maximum user engagement.

## Introduction

### Background

Social media platforms (SMPs), such as Twitter, Facebook, and Reddit, are commonly used by people to access health information. In the United States, 8 in 10 internet users access health information online, and 74% of these use SMPs. Meanwhile, public health agencies and pharmaceutical companies often use social media to engage with the public [1]. SMPs significantly contribute to the community by providing a communication platform for the public, patients, and health care professionals (HCPs) to talk about health concerns, eventually leading to better outcomes [2]. Additionally, SMPs also function as a medium to motivate patients by promoting health care education and providing the latest information to the community [1]. Analyzing social media content in the health care domain can reveal important dimensions, such as audience reach (eg, followers and subscribers), post source (eg, pharmaceutical companies, public health agencies), and post interactivity (eg, number of likes, retweets) [3]. A recent study discussed a machine learning (ML) approach to examining COVID-19 on Twitter [4]. Although it identifies discussion themes, there is no research on understanding the content shared by public health agencies and private organizations.

### Related Works

The positive impacts of using SMPs by patients and HCPs have been previously discussed [5]. Patients feel empowered and develop positive relationships with their HCPs. For instance, Ventola [1] discussed SMPs as a tool to share and promote healthy habits, share information, and interact with the public. Li et al [6] presented an analysis of social media's impact on the public. Their research discusses public perceptions of health-related content being classified as true, debatable, or false; the study shows that people have a strong tendency to adopt collective opinions while sharing health-related statements on social media.

There are different topic-clustering and content analysis techniques available to identify the characteristics of stakeholders (eg, pharmaceutical companies’ tweets for drug information) on SMPs [7,8]. A previous study presented an overview of techniques used for sentiment analysis in health care [9]. The researchers discuss multiple lexicon-based and ML-based approaches. The previous discussion on pharmaceutical companies has focused on COVID-19 vaccine–related public opinions [10,11]. Using latent dirichlet allocation (LDA) and valence aware dictionary and sentiment reasoner (VADER), researchers have examined topics, trends, and sentiments over time [10].

Prior research work has also focused on the response of G7 leaders during COVID-19 on Twitter [12,13]. The research classified viral tweets into appropriate categories, the most common being *informative*. Furthermore, researchers have recently presented a discussion on the harms and benefits of using Twitter during COVID-19 [14]. An epidemiological study conducted in 2020 investigated the news-sharing behavior on Twitter. Although it concluded that tweets that include news articles sharing pandemic information are popular, they cannot substitute public health agencies, organizations, or HCPs [15]. In addition, the study of public sentiments via artificial intelligence (AI) can provide a way to frame public health policies [16].

COVID-19 led to a rapid change in public sentiments over a short span of time [17]. People expressed sentiments of joy and gratitude toward good health and sadness and anger at the loss of life and stay-at-home orders [17,18]. Understanding public perceptions toward health-related content is important. Although the majority of people have a positive attitude toward social media, some feel more attention is required to promote the credibility of shared information [19]. Attempts have been made to capture peoples’ reactions to the pandemic; however, they are limited in scope. One study investigated the concerns originating toward public health interventions in North America via topic modeling [20], while another examined the role of beliefs and susceptibility information in public engagement on Twitter [21]. Statistical analysis also shows that health care organizations have to come forward to engage more with consumers [22]. The importance of risk communication strategies while using SMPs cannot be undermined [23].

Although a tweet’s engagement and sentiment can only be calculated once it has been posted, forecasting presents a fascinating way to predict the sentiments beforehand. Time series–based strategies, such as autoregressive integrated moving average (ARIMA) and vector autoregressions (VAR), have been used for forecasting emotions from SMPs [24,25]. The seasonal autoregressive integrated moving average with exogenous factors (SARIMAX) model was recently used to gain insights into people’s current emotional state via sentiment nowcasting on Twitter [26].

ML and natural language processing (NLP) algorithms have been recently used in various instances; for example, Bayesian ridge and ridge regression models were used for emotion prediction and health care analysis on large-scale data sets [27,28]. The elastic net and lasso regression have been previously used for health care access management and information exchange [29,30], while linear regression, decision tree, and random forest models are commonly used for epidemic-level disease tracking [31]. Different regression boosting algorithms, such as AdaBoost, light gradient boost , and gradient boost, have also been used for disease outbreak prediction [31]. Prophet, a Python library package, was recently used for COVID-19 outbreak prediction [32].

### Objective

The implications of social media communication by HCPs have been extensively discussed [33,34]. Although they focus on the advantages and methods of extracting health- and disease-related content from social media, there is currently a lack of understanding of how social media usage by public health agencies, nongovernment organizations (NGOs), and pharmaceutical companies resonates with society. Additionally, the study of tweets’ sentiments can supplement existing models for generating content for future tweets. Predicting the tweet sentiment is 1 way to achieve this goal. Therefore, it is crucial to convert this textual content into information for formulating future strategies and gaining valuable insights into perceptions of social media users.

The remainder of the paper is structured as follows: First, a preliminary analysis of topic modeling using the best-performing clustering algorithm is presented in the Methods section, followed by sentiment and engagement analysis using CardiffNLP’s *twitter-roberta-base-sentiment* model. We then conducted time series–based sentiment forecasting using 16 univariate models on the complete data set. The Results section outlines model topics obtained, which were used for generating heatmaps to obtain insights into topicwise tweets. Next, we discussed user engagement with its impact to understand whether there were specific occurrences of higher levels of engagement impacted by any offline events. In addition, we discussed results from best-performing sentiment-forecasting models. Finally, in the Discussion section, we draw conclusions and present an outline for future work.

## Methods

### Data Set

The data for this study (181,469 tweets) were gathered from the accounts of major US and Canadian health care organizations, pharmaceutical companies, and the World Health Organization (WHO) using the Twitter Academic API for Research v2 [35] during the time frame of January 1, 2017, to December 31, 2021. The top 5 pharmaceutical companies were selected based on the recommendations made by HCPs on Twitter [36]. Table 1 lists the number of tweets scraped for each Twitter handle. Each organization is referred to as a *user*, and the type of organization (ie, pharmaceutical company, public health agency, NGO) is referred to as a user group for the scope of this study.

**Figure 1:**
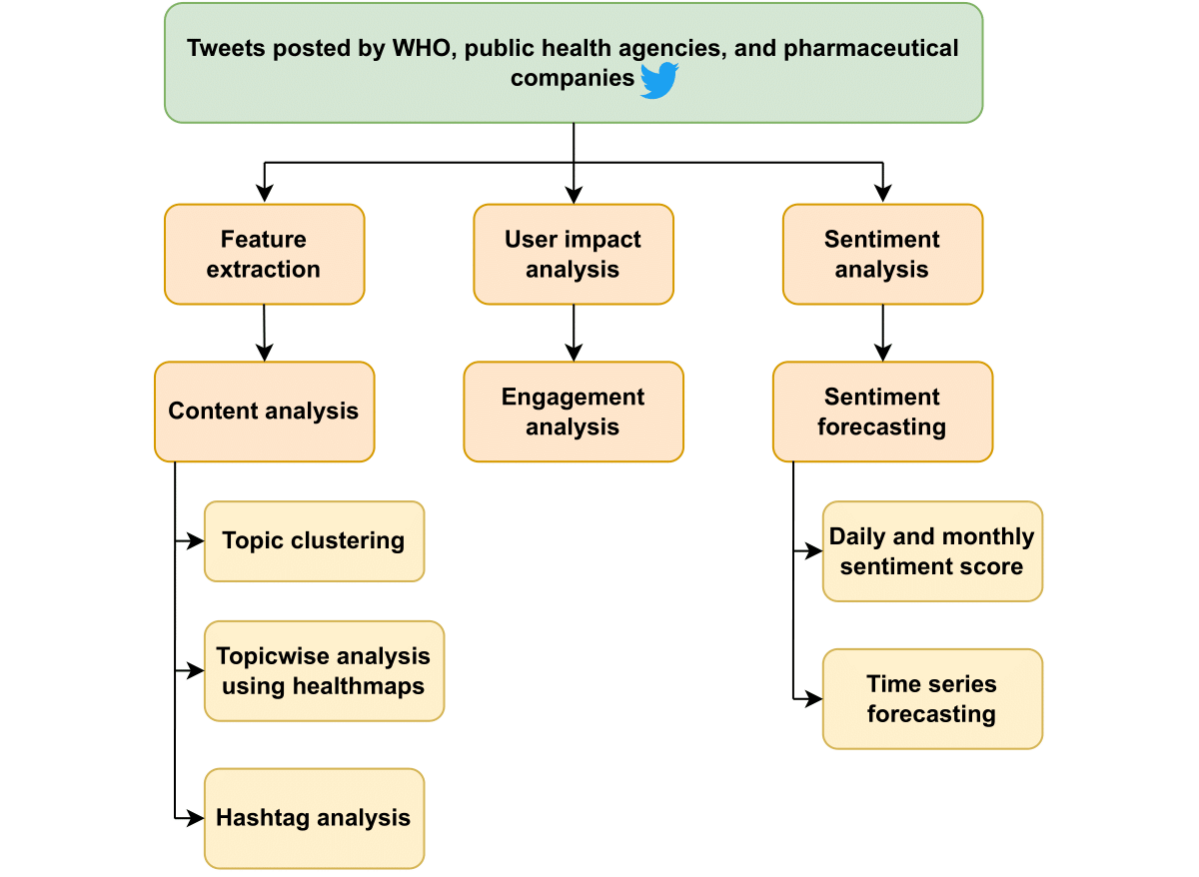
Overall research framework. WHO: World Health Organization.

The complete timeline was divided into 2 phases for analysis, *before* COVID-19 and *during* COVID-19, based on the confirmation of the first COVID-19 community transmission case in North America on February 26, 2020 [37]. Figure 1 presents an overview of the research framework.

**Table 1:** Distribution of tweets for the selected user accounts of 3 types of organizations.

Name of organization (Twitter handle)		Before COVID-19, n (%)	During COVID-19, n (%)	Total tweets, N
**Public health agencies**				
	Centers for Disease Control and Prevention (CDCgov)	8435 (58.6)	5963 (41.4)	14,398
	Centers for Disease Control and Prevention (CDC_eHealth)	1376 (86.3)	219 (13.7)	1594
	Government of Canada for Indigenous (GCIndigenous)	3505 (54.0)	2989 (46.0)	6494
	Health Canada and PHAC (GovCanHealth)	7878 (17.2)	37,907 (82.8)	45,785
	US Department of Health & Human Services (HHSGov)	7890 (56.9)	5969 (43.1)	13,859
	Indian Health Service (IHSgov)	1090 (44.7)	1346 (55.3)	2436
	Canadian Food Inspection Agency (InspectionCan)	4145 (62.2)	2516 (37.8)	6661
	National Institutes of Health (NIH)	5837 (71.6)	2314 (28.4)	8151
	National Indian Health Board (NIHB1)	1247 (51.1)	1195 (48.9)	2442
	US Food and Drug Administration (US_FDA)	5810 (59.7)	3925 (40.3)	9735
	Total	47,213 (42.3)	64,343 (57.7)	111,555
**Pharmaceutical companies**				
	AstraZeneca (AstraZeneca)	3462 (78.2)	963 (21.8)	4425
	Biogen (biogen)	1819 (61.9)	1120 (38.1)	2939
	Glaxo SmithKline (GSK)	4200 (69.3)	1857 (30.7)	6057
	Johnson & Johnson (JNJNews)	4813 (71.4)	1926 (28.6)	6739
	Pfizer (pfizer)	3637 (64.1)	2039 (35.9)	5676
	Total	17,931 (69.4)	7905 (30.6)	25,836
**NGO^a^**				
	World Health Organization (WHO)	24,775 (56.2)	19,303 (43.8)	44,078

^a^NGO: nongovernment organization.

### Content Analysis

The content of each user was divided into 2 phases, before and during COVID-19. We performed topic modeling on the tweets authored by the organizations by using the topics yielded by the best-performing topic model in order to explore the most and least talked about topics with the help of heatmaps. Additionally, we examined the top 10 hashtags used by these organizations.

#### Preprocessing

First, all nonalphabets (numbers, punctuation, new-line characters, and extra spaces) and Uniform Resource Locators (URLs) were removed using the regular expression module (*re 2.2.1*) [38] for all tweets. The cleaned text was then tokenized using the *nltk 3.2.5* library [39]. Next, stopwords were removed, followed by stemming using PorterStemmer, and lemmatizing using the WordNetLemmatizer from *nltk*.

#### Topic Modeling

Researchers have used term frequency–inverse document frequency (TF-IDF) to create document embeddings for tweets [40]. Following their approach, we preprocessed and generated document embeddings for tweets and input them to 5 different clustering algorithms: LDA, parallel LDA, nonnegative matrix factorization (NMF), latent semantic indexing (LSI), and the hierarchical dirichlet process (HDP). These clustering algorithms were executed 5 times with varying random seed values. The seed values accounted for the short and noisy nature of tweets. We calculated the coherence scores of the topic models, c_umass_ [41] and c_v_ [42], to confirm performance consistency over multiple runs.

We used Gensim LDA [43], Gensim LDA multicore (parallel LDA) [44], and Gensim LSI [44,45] models. For NMF and HDP models, we used online NMF for large corpora [46] and online variational inference [46,47] models, respectively.

#### Heatmaps

Heatmaps were generated using *seaborn* to analyze the volume of tweets for each topic. The topics yielded by the best-performing topic model as per the time phase (ie, before and during COVID-19) were leveraged to generate heatmaps. Each cell represented the total count of tweets for a particular topic by an organization. For example, among pharmaceutical companies, AstraZeneca had the highest number of tweets (n=1729, 49.9%) before COVID-19 for chronic diseases.

#### Hashtags

The top 10 hashtags mentioned in the users’ tweets were evaluated using the *advertools 0.13.0* module [48]. This tool extracts hashtags in social media posts. It was used for analyzing the similarities and differences in the tweeting behavior before and during COVID-19 and conducting topic analysis.

### Sentiment Analysis

Sentiment analysis is an NLP approach used to categorize the sentiments appearing in Twitter messages based on the keywords used in each tweet. We tested different models that classify a user’s tweet in 1 of 3 categories: positive, negative, and neutral. Although there is no common threshold for how many tweets should be sampled, we witnessed a range of around 2000 tweets [49-51] to several thousand tweets [52-54] when testing a model. For this study, we sampled 3000 tweets uniformly distributed over the span of our data collection time frame and from all Twitter handles. The tweets were then labeled by 3 distinct annotators, and the sentiment category with the highest votes was chosen as the overall sentiment. CardiffNLP’s *twitter-roberta-base-sentiment* model [55], which is trained on a 60 million Twitter corpus, was used to obtain sentiment labels on the sampled data set. We checked for similarity between human annotations and model labels, and the similarity percentage for CardiffNLP’s model was 69.96%; the model was therefore used to predict the sentiment on the remaining tweets of the users.

### Engagement Analysis

For a given user, Twitter defines the engagement rate [56] as presented in Equation (1):



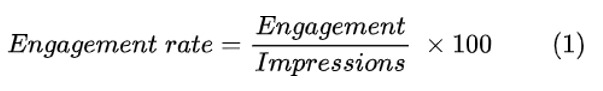



where “*Engagement* is the summation of the number of likes, replies, retweets, media views, tweet expansion, profile, hashtag, URL clicks, and new followers gained for every tweet, and *Impressions* is the total number of times a tweet has been seen on Twitter, such as through a follower’s timeline, Twitter search, or as a result of someone liking your tweet.”

Researchers have analyzed the impact (popularity) of Twitter handles by proposing heuristic and neural network–based models [57-59]. We defined it as a function of followers, following, the total number of tweets, and the profile age and calculated it using Equation (2):



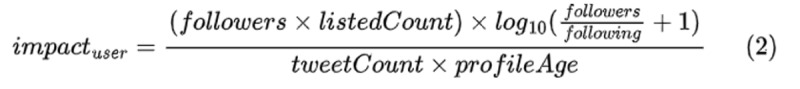



where *listedCount* is the number of public lists of which this user is a member.

The total number of tweets produced by a user was considered inversely proportional to the user’s impact, because a user tweeting occasionally and receiving higher engagement is more impactful than a user tweeting regularly with lower engagement.

Engagement analysis was performed to quantify the popularity of a topic generated. The engagement for each user was defined as the product of average engagement per day and their impact, as described in Equation (3). The average engagement per day was calculated as the sum of the count of likes, replies, retweets, and quotes per day. These reactions were aggregated from January 1, 2017, to December 31, 2021.



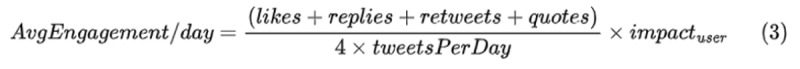



The exponential moving average (EMA) was calculated with a window span of 151 days for every user, and outliers were removed using the z-score, followed by smoothening of the average engagement per day to the eighth degree using the Savitzky-Golay filter [60].

### Sentiment Forecasting

To forecast the sentiment per day, we first needed to quantify the overall sentiment of the tweets from each user every day. We leveraged CardiffNLP’s *twitter-roberta-base-sentiment* model [55] to calculate the sentiments of all the tweets collected for our analysis and then calculated the daily sentiment score, as mentioned in Equation (4), based on the sentiment category with the maximum number of tweets for that day, followed by assigning the sentiment score based on the sentiment: 0 for *neutral* sentiment, the ratio of the count of positive tweets to total tweets for *positive* sentiment, and the negation of the ratio of the count of negative tweets to the total tweets for *negative* sentiment.



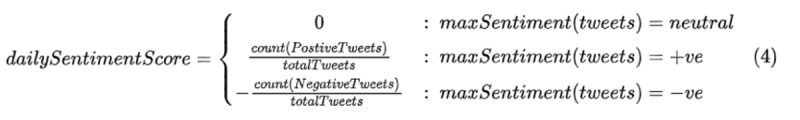



The daily sentiment scores were then resampled to a monthly mean sentiment score, which also helped us in handling missing values, if any. The complete timeline was divided into 2 phases (ie, before and during COVID-19), as discussed before, and the sentiment score was forecasted on 20% of the data set in each period for all user groups.

A grid search was used to find optimal hyperparameters, and 5-fold cross-validation was performed for every model. The *statsmodel* library [61] was used for ARIMA [62] and SARIMAX [63] models, and *pycaret* [64] was used for regression-based models. We also reported the performance of the *prophet* [65] model on the data set.

Three metrics, the mean absolute error (MAE), the mean square error (MSE), and the root-mean-square error (RMSE), were selected to evaluate the forecasting accuracy of the models. We considered 1-step-ahead forecasting for this study as it helped avoid problems related to cumulative errors from the preceding period.

### Computational Resources

The study was performed using Compute Canada (now called the Digital Research Alliance of Canada) resources, which provide access to advanced research computing (ARC), research data management (RDM), and research software (RS). The following is a list of the computing resources offered by one of the clusters from National Services (Digital Research Alliance), Graham:

- Central processing unit (CPU): 2x Intel E5-2683 v4 Broadwell@2.1 GHz
- Memory (RAM): 30 GB

## Results

### Content Analysis

The details of the parameters used for each model are discussed in Multimedia Appendix 1, Table S1. Table 2 shows the mean coherence scores (c_v_ and c_umass_) for each clustering algorithm. Although the HDP had the highest c_v_ scores in both time phases (ie, 0.696 and 0.650 before and during COVID-19, respectively), NMF had the best c_umass_ scores (–3.653 and –3.794, respectively) and generated the most meaningful topics for the data set (see Multimedia Appendix 1, Tables S2 and S3). Therefore, the top 5 topics generated by NMF were selected to search for on the first page of Google Search results. The resulting contents were then retrieved to interpret the extracted topic keywords to propose a suitable topic name. For example, for the set of keywords yielded by the topic model “*community health*, *care*, *community health services*, *health center*, *family health centers*, *community plan*, *community clinic*, *family health care*, *qualified health centers*, *health services*,” we assigned the topic *community health care*.

**Figure 2:**
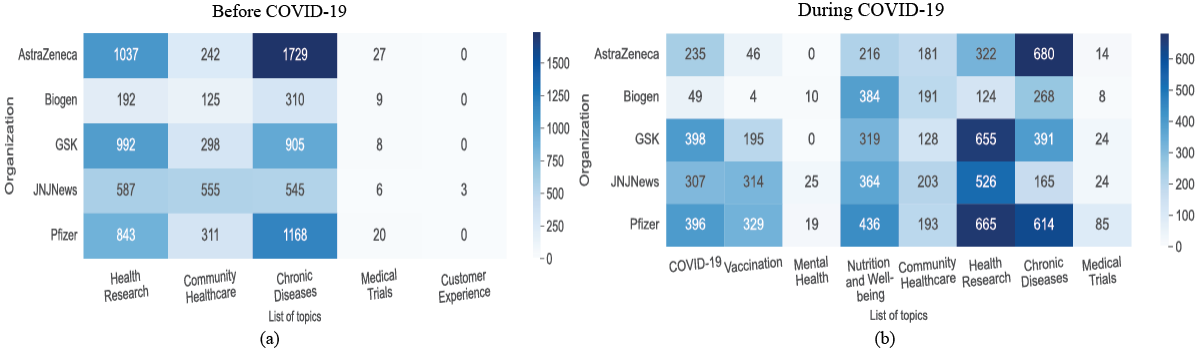
Scaled heatmaps showing topic distribution for pharmaceutical companies before and during COVID-19.

The scaled heatmaps showing the topic distribution for different Twitter handles are shown in Figure 2. Prior to COVID-19, chronic diseases were the most active topic, with a total of 9488 tweets from pharmaceutical companies and WHO (see Figure 2a). However, during COVID-19, we observed that COVID-19, health research, and chronic diseases were the most-discussed topics, with 52,148 tweets from all data sets combined (see Multimedia Appendix 1, Figures S1b and S1d).

**Figure 3:**
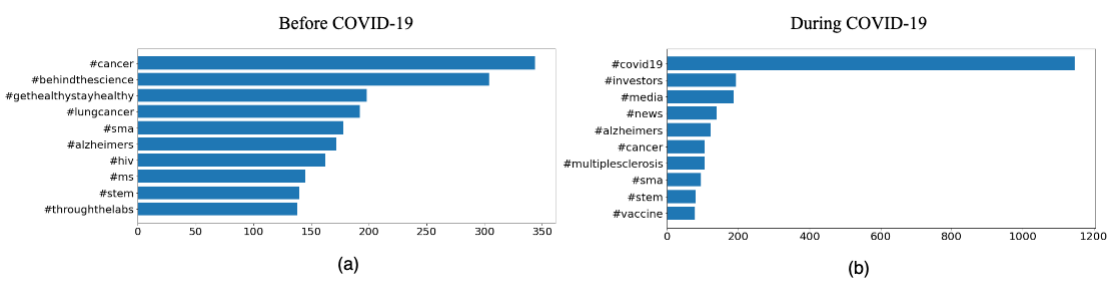
Top hashtags of pharmaceutical companies before and during COVID-19.

This shift in the tweets’ content was observed across the complete data set, and we further made the following inferences:

- Before COVID-19: Chronic diseases were the most talked about topic for pharmaceutical companies (AstraZeneca, 1729, 49.9%, tweets; Pfizer, 1168, 32.1%, tweets) and for WHO (4831, 19.5%, tweets), followed by tweets on health research (WHO, 1703, 6.9%, tweets; AstraZeneca, 1037, 29.9%, tweets). This is supported by Figure 3a, which shows #cancer, #lungcancer, #alzheimers, #hiv, and #ms to be prominently used in tweets. Among public health agencies, the NIH’s and the CDC’s Twitter handles were the most active, with 1840 (31.6%) and 1742 (20.6%) tweets discussing health research and chronic diseases, respectively, strongly supported by the most used hashtags #nativehealth and #foodsafety (refer to Multimedia Appendix 1, Figures S2a and S2c).
- During COVID-19: Chronic diseases and health research were the most active topics for AstraZeneca (680, 70.6%, tweets) and Glaxo SmithKline (GSK, 655, 35.2%, tweets), respectively. In addition, COVID-19 and vaccination were most talked about by GSK (398, 21.4%, tweets) and Pfizer (396, 19.4%, tweets). Figure 3b shows the hashtags supporting this: #covid19, #alzheimers, #cancer, #multiplesclerosis, and #vaccine. GovCanHealth was by far the most active public health agency on Twitter, with 16,832 (87.2%) tweets on health research, 16,449 (85.2%) tweets on vaccination, and 14,260 (73.8%) tweets on COVID-19, having #covid19, #coronavirus, and #covidvaccine as trending hashtags. The majority of the tweets by WHO were on COVID-19 (8911 tweets) and vaccination (2131 tweets), with #covid19, #coronavirus, and #vaccineequity appearing frequently in the tweets (refer to Multimedia Appendix 1, Figure S2d).

**Table 2:** Mean coherence scores and CPU^a^ time for different clustering algorithms.

Clustering algorithm		c_v_	c_umass_	Time taken (minutes:seconds)
**Before COVID-19**				
	LDA^b^	0.352	–5.526	17:11
	Parallel LDA	0.396	–3.709	5:48
	NMF^c^	0.493	–3.653	7:38
	LSI^d^	0.316	–5.921	0:16
	HDP^e^	0.696	–18.668	3:24
**During COVID-19**				
	LDA	0.456	–5.688	14:01
	Parallel LDA	0.446	–3.990	6:08
	NMF	0.567	–3.794	7:04
	LSI	0.381	–5.356	0:16
	HDP	0.650	–17.610	3:01

^a^CPU: central processing unit.

^b^LDA: latent dirichlet allocation.

^c^NMF: nonnegative matrix factorization.

^d^LSI: latent semantic indexing.

^e^HDP: hierarchical dirichlet process.

### Engagement Analysis

**Figure 4:**
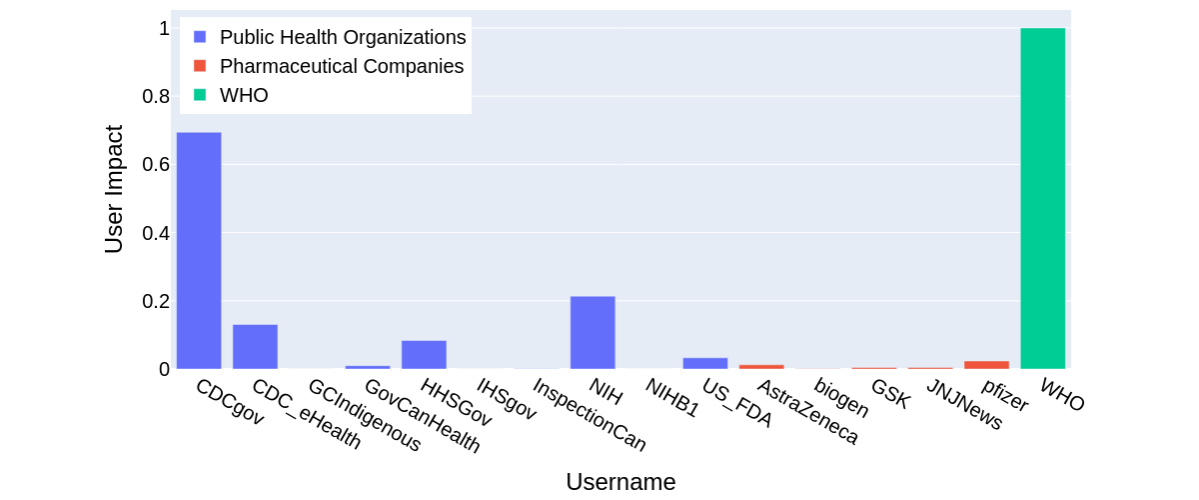
User impact of all Twitter handles scaled between 0 and 1. CDC: Centers for Disease Control and Prevention; NIH: National Institutes of Health; WHO: World Health Organization.

WHO (user impact=4171.24) had the highest impact overall, followed by public health agencies (CDC user impact=2895.87; NIH user impact=891.06). Among pharmaceutical companies, Pfizer’s user impact was the highest at 97.79. The user impact was normalized between the range of 0 and 1 and is shown in Figure 4.

**Figure 5:**
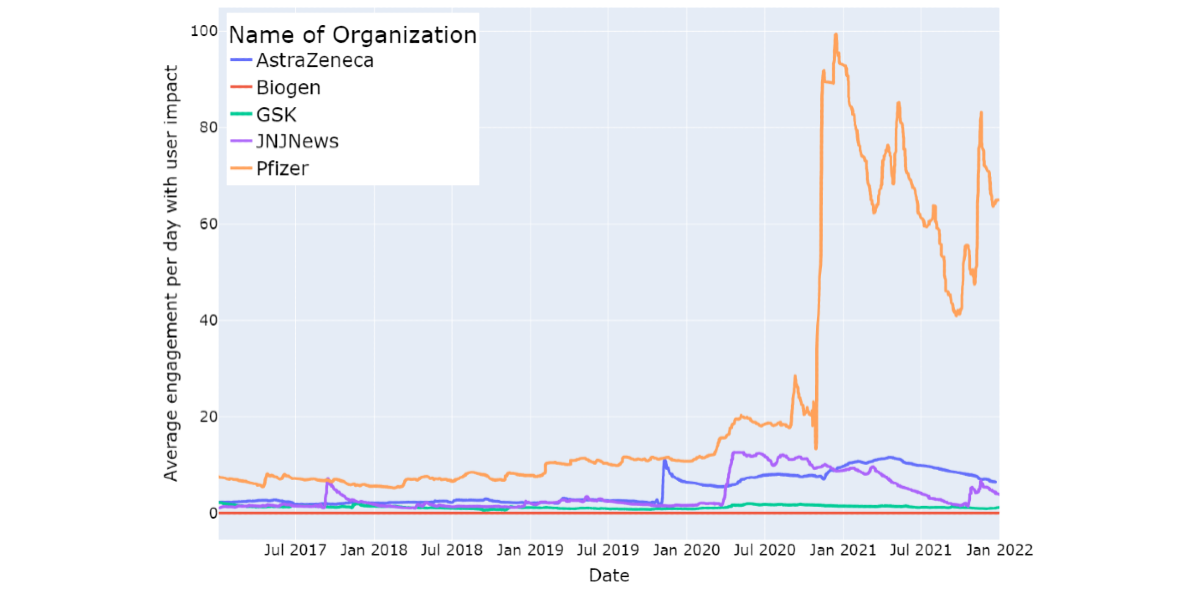
User engagement on Twitter accounts of pharmaceutical companies from January 1, 2017, to December 31, 2021.

Among pharmaceutical companies, Pfizer’s user engagement was far higher than that of others (Figure 5), both before and during COVID-19, with the highest engagement observed at the time of its COVID-19 vaccine’s success in November 2020. A jump in engagement was also observed in May 2021, when Pfizer announced its plan for helping India fight the second wave of coronavirus (refer to Multimedia Appendix 1, Table S4).

A similar trend was observed in public health agencies, with the CDC’s account showing the highest user engagement between March and June 2020, the early months of the COVID-19 pandemic. A sharp rise in user engagement was observed in May 2021, when the CDC announced a relaxation on social distancing and masking rules for fully vaccinated individuals. The user engagement on WHO’s account varied significantly over time. Its engagement was the highest in the time frame of February-April 2020, the early months of the pandemic, similar to what was observed for public health agencies. A sharp increase was seen in October 2020 following the announcement of the World Mental Health Day and in late 2020, when WHO made an announcement for COVID-19 vaccine development (refer to Multimedia Appendix 1, Figure S3).

### Sentiment Forecasting

Table 3 shows the MAE, MSE, and RMSE for the 16 models used on the data sets. Overall, ARIMA (univariate) and SARIMAX models performed best on the majority of the subsets of the data (divided as per the organization and period), and we further made the following inferences:

- Before COVID-19: ARIMA and SARIMAX models generated the lowest MSE (0.005) and RMSE (0.072) for pharmaceutical companies. When measuring the model performance through the MAE, ARIMA performed better than all other models (0.063). A similar trend was observed for public health agencies, with ARIMA having the lowest MAE (0.027) and SARIMAX having the lowest RMSE (0.031) and a tie between them for the MSE (0.001). SARIMAX had the lowest MAE (0.054), MSE (0.004), and RMSE (0.080) on the WHO data set.
- During COVID-19: Using the CatBoost regressor gave the lowest MAE (0.072) and RMSE (0.086), while the K-neighbors regressor yielded the lowest MSE (0.008) for pharmaceutical companies. Performing regression using AdaBoost generated the lowest MAE (0.084) and RMSE (0.105) among all models used, and SARIMAX had the lowest MSE (0.011) for public health agencies. For WHO, the elastic net, lasso regression, and light gradient boosting performed equally well, with all 3 models having the same MAE (0.046) and RMSE (0.059), and SARIMAX had the lowest MSE (0.004).

**Figure 6:**
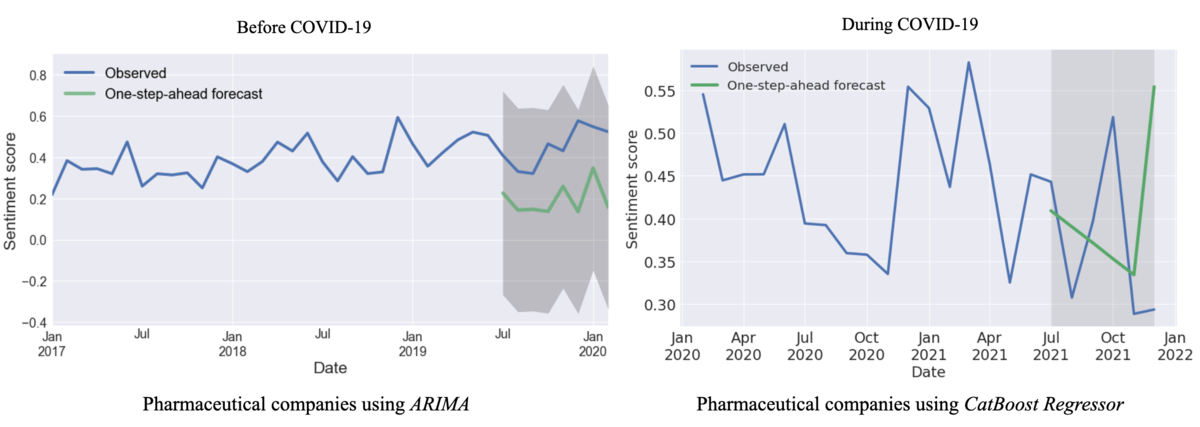
One-step-ahead forecast for all pharmaceutical companies before and during COVID-19 using the best-performing models from Table S1 (Multimedia Appendix 1). ARIMA: autoregressive integrated moving average.

Figure 6a shows the 1-step-ahead forecast for pharmaceutical companies before COVID-19 using ARIMA. The model was trained on sentiment scores from January 2017 to June 2019 and tested on data from July 2019 to February 2020 for tweets before COVID-19. The 1-step-ahead forecasting aligned well with the observed sentiment scores, and we obtained similar results for public health agencies and WHO. The organizations showed some deviations from observed sentiments while conducting 1-step-ahead forecasting during COVID-19, making it difficult to predict their sentiment accurately, as seen in Multimedia Appendix 1, Figure S4.

To verify the forecasting performance of these models, we checked for the nature of their residual errors (ie, whether the residuals of the models were normally distributed with mean 0 and SD 1 and were uncorrelated). From Multimedia Appendix 1, Figure S5, as in the case of public health agencies, before COVID-19 using ARIMA, we confirmed the aforementioned through *plot_diagnostics*. The green kernel density estimation (KDE) line closely followed the normal distribution (N ∊ {0,1}) line in the top-right corner of Multimedia Appendix 1, Figure S5, which is a positive indicator that the residuals were scattered normally. The quantile-quantile (Q-Q) plot on the bottom left shows that the distribution of residuals (blue dots) approximately followed the linear trend of samples drawn from a standard normal distribution, N. This confirms again that the residuals were normally distributed. The residuals over time (top left in Multimedia Appendix 1, Figure S5) showed no apparent seasonality and have 0 mean. The autocorrelation plot (ie, correlogram) attested this, indicating that the time series residuals exhibited minimal correlation with lagged forms of themselves. Thus, these findings encouraged us to believe that our models provide an adequate fit, which might aid us in understanding the sentiments of the organizations and forecasting their values without overburdening our hardware with computationally heavy models.

**Table 3:** Results of time series sentiment forecasting using different ML^a^ models (all metrics are 5-fold cross-validation).

Models	Pharmaceutical companies								Public health agencies									WHO^b^						
	Before COVID-19			During COVID-19				Before COVID-19					During COVID-19				Before COVID-19					During COVID-19		
	MAE^c^	MSE^d^	RMSE^e^	MAE	MSE	RMSE	MAE			MSE	RMSE	MAE		MSE	RMSE	MAE			MSE	RMSE	MAE		MSE	RMSE
ARIMA^f^	0.063^g^	0.005^g^	0.072^g^	0.098	0.013	0.112	0.027^g^			0.001^g^	0.032^h^	0.240		0.082	0.286	0.066^h^			0.006^h^	0.080^h^	0.106		0.012	0.111
SARIMAX^i^	0.065^h^	0.005^g^	0.072^g^	0.084	0.011	0.104	0.028^j^			0.001^g^	0.031^g^	0.709		0.011^g^	0.106^h^	0.054^g^			0.004^g^	0.061^g^	0.047^h^		0.004^g^	0.066
Bayesian ridge	0.083	0.010	0.100	0.102	0.018	0.119	0.031			0.001	0.037	0.141		0.037	0.163	0.075^j^			0.009^j^	0.087^j^	0.061		0.008	0.075
Ridge regression	0.069	0.008	0.085	0.079	0.011	0.094	0.030			0.002	0.038	0.124		0.029	0.147	0.076			0.009	0.091	0.056		0.007	0.068
CatBoost regressor	0.066	0.007^j^	0.080^h^	0.072^g^	0.008^h^	0.086^g^	0.027^h^			0.001^h^	0.035	0.104		0.023	0.127	0.079			0.009	0.089	0.052		0.007	0.065
K-neighbors regressor	0.070	0.009	0.087	0.075^h^	0.008^g^	0.087^h^	0.030			0.001	0.036	0.093^j^		0.022	0.113	0.081			0.011	0.100	0.050		0.007	0.061^j^
Elastic net	0.070	0.008	0.088	0.080	0.009^j^	0.093^j^	0.029			0.001^h^	0.035	0.087^h^		0.021^j^	0.109^j^	0.082			0.011	0.100	0.046^g^		0.006^h^	0.059^g^
Lasso regression	0.070	0.008	0.088	0.080	0.009^j^	0.093^j^	0.029			0.001	0.035	0.087^h^		0.021^j^	0.109^j^	0.082			0.011	0.100	0.046^g^		0.006^h^	0.059^g^
Random forest regressor	0.065^j^	0.007^h^	0.081^j^	0.080	0.010	0.093	0.028			0.001^h^	0.034^j^	0.110		0.024	0.134	0.082			0.009	0.090	0.047^j^		0.006^j^	0.060^h^
Light gradient boosting machine	0.070	0.008	0.088	0.080	0.009^j^	0.093^j^	0.029			0.001^h^	0.035	0.087^h^		0.021^j^	0.109^j^	0.082			0.011	0.100	0.046^g^		0.006^h^	0.059^g^
Gradient boosting regressor	0.075	0.008	0.086	0.079	0.010	0.094	0.029			0.001^j^	0.036	0.141		0.034	0.168	0.082			0.010	0.094	0.051		0.008	0.064
AdaBoost regressor	0.070	0.007	0.082	0.080	0.010	0.091	0.029			0.001	0.037	0.084^g^		0.020^h^	0.105^g^	0.087			0.010	0.096	0.057		0.007	0.072
Extreme gradient boosting	0.068	0.009	0.087	0.080	0.011	0.098	0.031			0.002	0.040	0.151		0.045	0.171	0.087			0.011	0.098	0.055		0.007	0.065
Decision tree regressor	0.076	0.009	0.086	0.087	0.013	0.106	0.029			0.001	0.037	0.112		0.030	0.142	0.098			0.014	0.111	0.048		0.006^j^	0.061
Linear regression	0.245	0.312	0.314	0.094	0.017	0.114	0.157			0.164	0.216	0.124		0.029	0.148	2.367			52.719	3.334	0.062		0.008	0.076
Prophet	0.108	0.016	0.126	0.089	0.011	0.104	0.040			0.002	0.049	0.120		0.015	0.124	0.114			0.020	0.143	0.086		0.011	0.106

^a^ML: machine learning.

^b^WHO: World Health Organization.

^c^MAE: mean absolute error.

^d^MSE: mean squared error.

^e^RMSE: root-mean-square error.

^f^ARIMA: autoregressive integrated moving average.

^g^The highest-performing forecasting method.

^h^The second-highest-performing forecasting method.

^i^SARIMAX: seasonal autoregressive integrated moving average with exogenous factors.

^j^The third-highest-performing forecasting method.

## Discussion

### Principal Findings

In this paper, we proposed a framework for using NLP-based text-mining techniques for performing comprehensive social media content analysis of various health care organizations. We processed reasonably large amounts of textual data for topic modeling, sentiment and engagement analysis, and sentiment forecasting. Our study revealed the following key findings:

- Being the most active organization on social media does not translate to more user impact. WHO and the US public health agency CDC generated far more user impact than the Public Health Agency of Canada, even though the latter had a high number of relevant tweets when analyzed topicwise. People are more likely to engage with *neutral* tweets, which usually consist of some public health announcement rather than exclusively *positive* or *negative* tweets. This might mean that organizations can leverage this knowledge while creating content for social media posts in the future to increase their visibility in the online sphere.
- Certain topics normally translate to more user engagement. Although the content on chronic diseases and health research dominated most of the tweets posted over the study period, there was a marked shift toward a discussion on COVID-19 and vaccination for public health agencies, more than what was observed in pharmaceutical companies. Tweets on COVID-19 and chronic diseases generate more interest among the public. Perhaps surprisingly, we found that people are not much receptive to content on medical trials, often shared by pharmaceutical companies, unless it concerns a public health emergency, such as the COVID-19 pandemic. Using particular hashtags certainly helps in generating engagement, as we found that most user engagement was highly skewed toward tweets concerning COVID-19. Moreover, our study revealed that compared to the user engagement patterns found in the majority of health care organizations (ie, with peaks observed around major events or announcements), there are wide variations in user engagement for WHO. This could be due to the global presence of WHO, implying that it might not be the same set of followers engaging with its content every time, but rather only those who are impacted by or interested in the content in some way.
- When the content is structured, results tend to exceed expectations. We conducted sentiment forecasting on the data sets using different moving averages and various ML univariate models. Surprisingly, we observed that when the content is structured, as is normally the case for that available on official Twitter accounts, results tend to exceed expectations, more so before COVID-19 than during COVID-19. The models used in this research are able to predict monthwise tweet sentiment with high accuracy and low errors. This helped us in analyzing our work in-depth, and we did not need to create any multivariate ML models. Results show that commonly used ARIMA and SARIMAX models work well, and they can be used for predicting tweet sentiments on live data. This could also help organizations correlate tweet sentiment with user engagement. For example, the highest engagement on Pfizer’s tweets was for the ones labeled *neutral*, implying that the organization should structure the content of its future tweets in a similar manner to maintain higher levels of engagement. Furthermore, tweets that mention more news-relevant content might be able to translate it into more user engagement.

### Limitations and Future Work

There are 3 limitations of this study that could be addressed in future research. First, this work focused on dividing the tweets into 2 phases, *before* and *during* COVID-19. In the future, researchers can pursue other methods of structuring the analysis timeline. Second, this study dealt with only the structured textual content of tweets. It would be interesting to also incorporate the presence of image attributes in future studies. Finally, as the scope of this study was limited to health care organizations, we did not account for public demographics. Understanding the demographic background of the public engaging with this content is another area that can be explored in future studies.

### Conclusion

This study examined the online activity of US and Canadian health care organizations on Twitter. The NLP-based analysis of social media presented here can be incorporated to gauge engagement on the previously published tweets and to generate tweets that create an impact on people accessing health information via SMPs. As organizations continue to leverage SMPs by providing the latest information to the community, predicting a tweet’s sentiment before publishing can boost an organization’s perception by the public. In conclusion, we found that performing content analysis and sentiment forecasting on an organization’s social media usage provides a comprehensive view of how it resonates with society.

## Appendix

Multimedia Appendix 1 Topics and user engagement.

## Abbreviations

ARC: advanced research computing
ARIMA: autoregressive integrated moving average
CDC: Centers for Disease Control and Prevention
CPU: central processing unit
HCP: health care professional
HDP: hierarchical dirichlet process
LDA: latent dirichlet allocation
LSI: latent semantic indexing
MAE: mean absolute error
ML: machine learning
MSE: mean squared error
NGO: nongovernment organization
NIH: National Institutes of Health
NLP: natural language processing
NMF: nonnegative matrix factorization
RMSE: root-mean-square error
SARIMAX: seasonal autoregressive integrated moving average with exogenous factors
SMP: social media platform
TF-IDF: term frequency–inverse document frequency
WHO: World Health Organization

## References

[1] Ventola CL (2014). Social media and health care professionals: benefits, risks, and best practices. P T.

[2] Househ M (2013). The use of social media in healthcare: organizational, clinical, and patient perspectives. Stud Health Technol Inform.

[3] Zhou L, Zhang D, Yang CC, Wang Y (2018). Harnessing social media for health information management. Electron Commer Res Appl.

[4] Xue J, Chen J, Hu R, Chen C, Zheng C, Su Y, Zhu T (2020). Twitter discussions and emotions about the COVID-19 pandemic: machine learning approach. J Med Internet Res.

[5] Benetoli A, Chen T, Aslani P (2018). How patients' use of social media impacts their interactions with healthcare professionals. Patient Educ Couns.

[6] Li H, Sakamoto Y (2014). Social impacts in social media: an examination of perceived truthfulness and sharing of information. Comput Hum Behav.

[7] Lu Y, Wu Y, Liu J, Li J, Zhang P (2017). Understanding health care social media use from different stakeholder perspectives: a content analysis of an online health community. J Med Internet Res.

[8] Tyrawski J, DeAndrea DC (2015). Pharmaceutical companies and their drugs on social media: a content analysis of drug information on popular social media sites. J Med Internet Res.

[9] Abualigah L, Alfar H, Shehab M, Abd Elaziz M, Al-qaness MAA, Ewees AA (2020). Sentiment analysis in healthcare: a brief review. Recent Advances in NLP: The Case of Arabic Language.

[10] Chandrasekaran R, Mehta V, Valkunde T, Moustakas E (2020). Topics, trends, and sentiments of tweets about the COVID-19 pandemic: temporal infoveillance study. J Med Internet Res.

[11] Poddar S, Mondal M, Misra J Winds of Change: Impact of COVID-19 on Vaccine-Related Opinions of Twitter Users.

[12] Rufai S, Bunce C (2020). World leaders' usage of Twitter in response to the COVID-19 pandemic: a content analysis. J Public Health (Oxf).

[13] Haman M (2020). The use of Twitter by state leaders and its impact on the public during the COVID-19 pandemic. Heliyon.

[14] Rosenberg H, Syed S, Rezaie S (2020). The Twitter pandemic: the critical role of Twitter in the dissemination of medical information and misinformation during the COVID-19 pandemic. CJEM.

[15] Park HW, Park S, Chong M (2020). Conversations and medical news frames on Twitter: infodemiological study on COVID-19 in South Korea. J Med Internet Res.

[16] Hussain A, Tahir A, Hussain Z, Sheikh Z, Gogate M, Dashtipour K, Ali A, Sheikh A (2021). Artificial intelligence-enabled analysis of public attitudes on Facebook and Twitter toward COVID-19 vaccines in the United Kingdom and the United States: observational study. J Med Internet Res.

[17] Lwin MO, Lu J, Sheldenkar A, Schulz PJ, Shin W, Gupta R, Yang Y (2020). Global sentiments surrounding the COVID-19 pandemic on Twitter: analysis of Twitter trends. JMIR Public Health Surveill.

[18] Dubey AD (2020). Twitter sentiment analysis during COVID19 Outbreak. SSRN Electron J.

[19] Gao S, He L, Chen Y, Li D, Lai K (2020). Public perception of artificial intelligence in medical care: content analysis of social media. J Med Internet Res.

[20] Jang H, Rempel E, Roth D, Carenini G, Janjua NZ (2021). Tracking COVID-19 discourse on Twitter in North America: infodemiology study using topic modeling and aspect-based sentiment analysis. J Med Internet Res.

[21] Tang L, Liu W, Thomas B, Tran HTN, Zou W, Zhang X, Zhi D (2021). Texas public agencies' tweets and public engagement during the COVID-19 pandemic: natural language processing approach. JMIR Public Health Surveill.

[22] Koumpouros Y, Toulias TL, Koumpouros N (2015). The importance of patient engagement and the use of social media marketing in healthcare. Technol Health Care.

[23] Slavik CE, Buttle C, Sturrock SL, Darlington JC, Yiannakoulias N (2021). Examining tweet content and engagement of Canadian public health agencies and decision makers during COVID-19: mixed methods analysis. J Med Internet Res.

[24] Tommasel A, Diaz-Pace A, Rodriguez JM, Godoy D (2021). Forecasting mental health and emotions based on social media expressions during the COVID-19 pandemic. Inf Discov Deliv.

[25] McClellan C, Ali MM, Mutter R, Kroutil L, Landwehr J (2017). Using social media to monitor mental health discussions - evidence from Twitter. J Am Med Inform Assoc.

[26] Miliou I, Pavlopoulos J, Papapetrou P (2021). Sentiment nowcasting during the COVID-19 pandemic. Discovery Science.

[27] Harper R, Southern J (2022). A Bayesian deep learning framework for end-to-end prediction of emotion from heartbeat. IEEE Trans Affective Comput.

[28] Deepa N, Prabadevi B, Maddikunta PK, Gadekallu TR, Baker T, Khan MA, Tariq U (2020). An AI-based intelligent system for healthcare analysis using Ridge-Adaline stochastic gradient descent classifier. J Supercomput.

[29] Barrera Ferro D, Brailsford S, Bravo C, Smith H (2020). Improving healthcare access management by predicting patient no-show behaviour. Decis Support Syst.

[30] Li Y, Vinzamuri B, Reddy CK (2014). Constrained elastic net based knowledge transfer for healthcare information exchange. Data Min Knowl Disc.

[31] Singh R, Singh R (2021). Applications of sentiment analysis and machine learning techniques in disease outbreak prediction – A review. Mater Today.

[32] Mengistie T (2020). COVID-19 outbreak data analysis and prediction modeling using data mining technique. Int J Comput.

[33] Denecke K, Nejdl W (2009). How valuable is medical social media data? Content analysis of the medical web. Inf Sci.

[34] Nawaz MS, Bilal M, Lali MI, Ul Mustafa R, Aslam W, Jajja S (2017). Effectiveness of social media data in healthcare communication. J Med Imaging Health Inform.

[35] Twitter API: Academic Research Access.

[36] Kangley M HCPs Discuss ‘Booster Shot’ to Decrease the High Spread of the Delta Variant.

[37] Jorden MA, Rudman SL, Villarino E, Hoferka S, Patel MT, Bemis K, Simmons CR, Jespersen M, Iberg Johnson J, Mytty E, Arends KD, Henderson JJ, Mathes RW, Weng CX, Duchin J, Lenahan J, Close N, Bedford T, Boeckh M, Chu HY, Englund JA, Famulare M, Nickerson DA, Rieder MJ, Shendure J, Starita LM, CDC COVID-19 Response Team (2020). Evidence for limited early spread of COVID-19 within the United States, January-February 2020. Morb Mortal Wkly Rep.

[38] PyPI regex 2022.7.9.

[39] PyPI nltk 3.7.

[40] Lilleberg J, Zhu Y, Zhang Y (2015). Support vector machines and Word2vec for text classification with semantic features.

[41] Newman D, Lau J, Grieser K (2010). Automatic evaluation of topic coherence. https://aclanthology.org/N10-1012.

[42] Röder M, Both A, Hinneburg A (2015). Exploring the space of topic coherence measures.

[43] Gemsim Latent Dirichlet Allocation.

[44] Gensim Parallelized Latent Dirichlet Allocation.

[45] Gensim Latent Semantic Indexing.

[46] Gensim Non-Negative Matrix Factorization.

[47] Gensim Hierarchical Dirichlet Process.

[48] PyPI advertools 0.13.1.

[49] Alomari K, ElSherif H, Shaalan K (2017). Arabic tweets sentimental analysis using machine learning. Advances in Artificial Intelligence: From Theory to Practice.

[50] Peisenieks J, Skadins R (2014). Uses of machine translation in the sentiment analysis of tweets.

[51] Şaşmaz E, Tek F (2021). Tweet sentiment analysis for cryptocurrencies.

[52] Golubev A, Loukachevitch N (2020). Improving results on Russian sentiment datasets. Communications in Computer and Information Science.

[53] Nabil M, Aly M, Atiya A (2015). ASTD: Arabic Sentiment Tweets Dataset.

[54] Rustam F, Khalid M, Aslam W, Rupapara V, Mehmood A, Choi GS (2021). A performance comparison of supervised machine learning models for Covid-19 tweets sentiment analysis. PLoS One.

[55] Hugging Face cardiffnlp / twitter-roberta-base-sentiment.

[56] About Your Activity Dashboard.

[57] Daniluk M, Dabrowski J, Rychalska B (2021). Synerise at RecSys 2021: Twitter user engagement prediction with a fast neural model.

[58] Razis G, Anagnostopoulos I (2014). InfluenceTracker: rating the impact of a Twitter account.

[59] Son J, Lee J, Oh O, Lee HK, Woo J (2020). Using a heuristic-systematic model to assess the Twitter user profile’s impact on disaster tweet credibility. Int J Inf Manag.

[60] Marinai S, Dengel A (2004). Document Analysis Systems VI: 6th International Workshop, DAS 2004, Florence, Italy, September 8-10, 2004, Proceedings.

[61] statsmodels.

[62] statsmodels.tsa.arima.model.ARIMA.

[63] statsmodels.tsa.statespace.sarimax.SARIMAX.

[64] PyPI pycaret.

[65] PyPI prophet.

