# Supplementary material for: Synergy Between Public and Private Health Care Organizations During COVID-19 on Twitter: Sentiment and Engagement Analysis Using Forecasting Models
Source: JMIR Med Inform. 2022 Aug 18;10(8):e37829. doi: 10.2196/37829 (PMC9390834; doi:10.2196/37829)
Supplement: Multimedia Appendix 1 [file medinform_v10i8e37829_app1.pdf]

Multimedia Appendix: Topics and User Engagement

Clustering Algorithm	Epochs	Chunk Size	Workers (Number of CPU cores)	Evaluation Period (seconds)	α (*A-priori* belief on document - topic distribution)	η (*A-priori* belief on topic - word distribution)	K (Gradient descent step size)	Minimum normalizing probability
LDA	50	1000	NA	10	0.01	0.9	NA	NA
Parallel LDA	50	1000	7	10	0.01	0.9	NA	NA
LSI	NA	1000	NA	NA	NA	NA	NA	NA
NMF	50	1000	NA	10	NA	NA	1	0
HDP	NA	1000	NA	NA	0.01	NA	1	NA

Table S1: Model parameters for topic clustering with TF-IDF document embeddings.

HDP	NMF
['commonwealth','speedy','multi-vitamin','vaccine', 'weather-wise','unopen','salmon','breadth','land', '#skincancerawarenessmonth']	['vaccine','disease','protect people','prevent death', 'cancer','research']
['prop','goldstein','mihcha','kezspm','age','open','mohmv', 'thisisdiabetic','onco']	['health for all','healthcare','community health', 'vaccines work']

Table S2: Sample of topic keywords generated using HDP and NMF

Time Phase	Topic	Topic Keywords
Before COVID-19	*Health Research*[a]	*['cancer','research','vaccine','advancements','find','medical research','national institute for health','nih research','icon pra','qualitative health research','mental health research','mhsrs','public health research','integrative medicine research','medical trials']*
	Community Healthcare[a]	*['community health','care','community health services','health center','family health centers','community plan','community clinic','family healthcare','qualified health centers','health services']*
	Chronic Diseases[a]	*['angina','arthritis','asthma','bipolar disorder','cancer','hypertension','stroke','COPD','diabetes','heart attack','sleep apnea','disease','chronic','lupus','multiplesclerosis','lung cancer','ovarian cancer','heart failure','kidney disease','breast cancer','prostate cancer','spinal disorder','hiv','hemophilia','pneumonia','malaria','aids','tb','tuberculosis']*

	Medical Trials[a]	*['clinical trials','medical trials','paid trials','medical research studies','cancer clinical trials','hydoxychloroquine studies','randomized clinical trial','applied clinical trials','oncology clinical trials','celerion clinical trials','alzheimer clinical trials','registered clinical trials','depression clinical trials','weight loss clinical trials','artificial kidney human trials']*
	Customer Experience	['connected customer','customer experience','customer journey','user journey','user happiness','client satisfaction','seamless experience','measuring customer experience']
During COVID-19	COVID-19	['covid 19', 'virus', 'coronavirus', 'covid 19 cases', 'covid 19 deaths','covid 19 passport', 'covid 19 insurance', 'quarantine', 'pandemic', 'outbreak', 'social distancing', 'self isolation', 'cases', 'deaths', 'infections', 'fatality rate', 'mortality', 'masks', 'hygiene', 'state of emergency', 'surveillance', 'infectivity', 'communicable disease', 'community spread','containment','epidemic','herd immunity','ppe','personal protective equipment','respirator','SPO2','severe acute respiratory syndrome','contact tracing','hydroxychloroquine','risk']
	Vaccination	['covaxin','vaccine','covid vaccine','mrna vaccine','vaccine finder','herd immunity','booster shot','vaccine appointment','mandatory vaccine','vaccination card','vaccination passport','vaccination rates','inoculation','covishield']
	Mental Health	['anxiety','bipolar disorder','depression','panic','ptsd','schizophrenia','sucidal ideation','suicide','alzheimers','parkinson','mental illness','mental health day','mental health counselor','mental health services','mental disorder','clinical psychologist','behavioral health','mental health awareness','mental health therapist','mhfa','mental disability','psychologist','family therapists','licensed clinical social worker','strong minds','mental health stigma','mental health resources']
	Nutrition and Well-being	['healthy living','community','support','helping','awareness','development','innovation','well being','nutrition','diet','healthy diet','skin fuel','eat well be healthy','understanding nutrition and well being',' good sleep','nutritious foods']
	Community Healthcare[a]	*['community health','care','community health services','health center','family health centers','community plan','community clinic','family healthcare','qualified health centers','health services']*
	Health Research[a]	*['cancer','research','vaccine','advancements','find','medical research','national institute for health','nih research','icon pra','qualitative health research','mental health research','mhsrs','public health research','integrative medicine research','medical trials']*
	Chronic Diseases[a]	*['angina','arthritis','asthma','bipolar disorder','cancer','hypertension','stroke','COPD','diabetes','heart attack','sleep apnea','disease','chronic','lupus','multiplesclerosis','lung cancer','ovarian cancer','heart failure','kidney disease','breast cancer','prostate cancer','spinal*

		disorder','hiv','hemophilia','pneumonia','malaria','aids','tb','tuberculosis']
	Medical Trials[a]	*['clinical trials','medical trials','paid trials','medical research studies','cancer clinical trials','hydoxychloroquine studies','randomized clinical trial','applied clinical trials','oncology clinical trials','celerion clinical trials','alzheimer clinical trials','registered clinical trials','depression clinical trials','weight loss clinical trials','artificial kidney human trials']*

Table S3: List of topics obtained using NMF model

Note[a]: *Italicized* topic keywords are repeated in both timeframes, *before COVID-19* and *during COVID-19*.

**Organization**	**Tweet ID**	**Created at**	**Tweet**	**Average user engagement/Average user engagement with impact**
Pfizer	1325767629890592771	2020-11-09 11:50:09+00:00	UPDATE: We are proud to announce, along with @BioNTech_Group, that our mRNA-based #vaccine candidate has, at an interim analysis, demonstrated initial evidence of efficacy against #COVID19 in participants without prior evidence of SARS-CoV-2 infection.	13,901.75/ 319.74
Pfizer	1389203084879011840	2021-05-03 13:00:00+00:00	Today we have announced we are mobilizing the largest humanitarian relief effort in our company's history to help the people of India fight the vicious second wave of coronavirus that is currently ravaging the nation. https://t.co/klVnkAjkcw	3,132.25/ 72.04
CDC	1392911350058323973	2021-05-13 18:35:19+00:00	UPDATE: If you are fully vaccinated against #COVID19, you can resume activities without wearing a mask or staying 6 feet apart, except where required by federal, state, local, tribal or territorial laws, incl. local business and workplace guidance. More: https://t.co/FJMon7WlFO	28,997.50/ 20,124.26
WHO	1313841832598687749	2020-10-07 14:01:17+00:00	We are thrilled to have @SuperM joining our Big Event for Mental Health on #WorldMentalHealthDay! Stay tuned for #SuperMxWHO! 📅This Saturday 10.10.2020<br>🕙10h00 EST<br>🕓16h00 CEST<br>🕚23h00 KST<br>More information:<br>▶️https://t.co/seFE6mb3O7	17,398.00/ 17,398.00

			#SuperM https://t.co/zfYaJfr6nn	

Table S4: Selected tweets having high user engagement

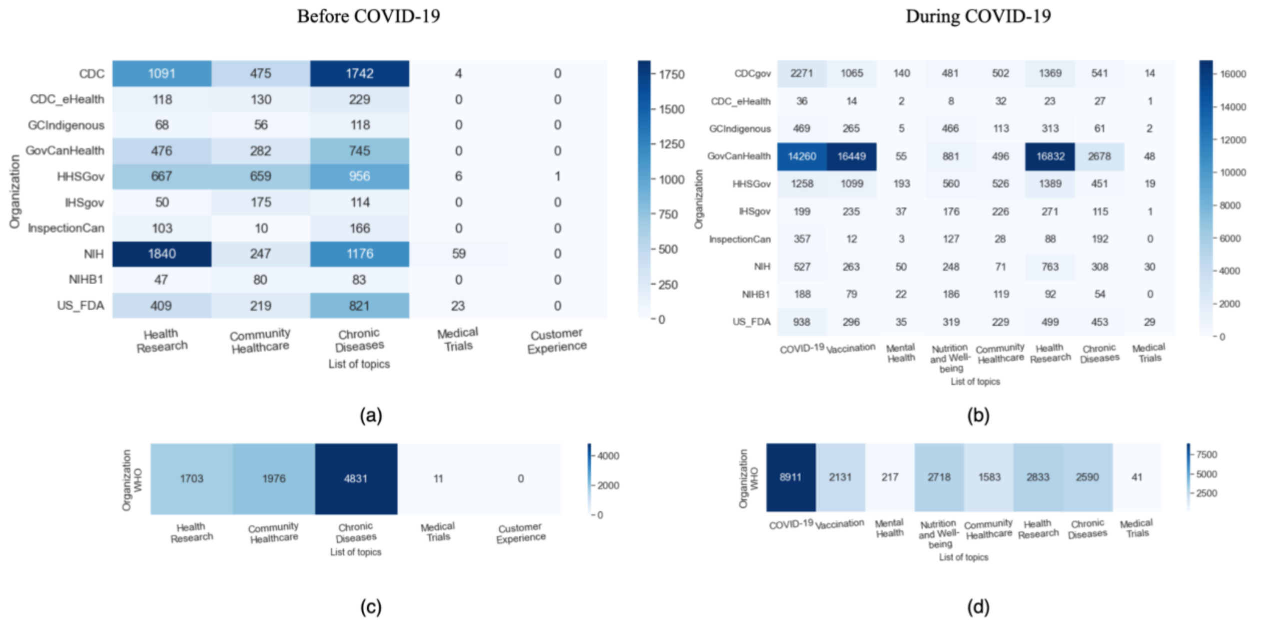

Figure S1: Scaled heatmaps showing topic distribution for Public Health Agencies and WHO before COVID-19 and during COVID-19.

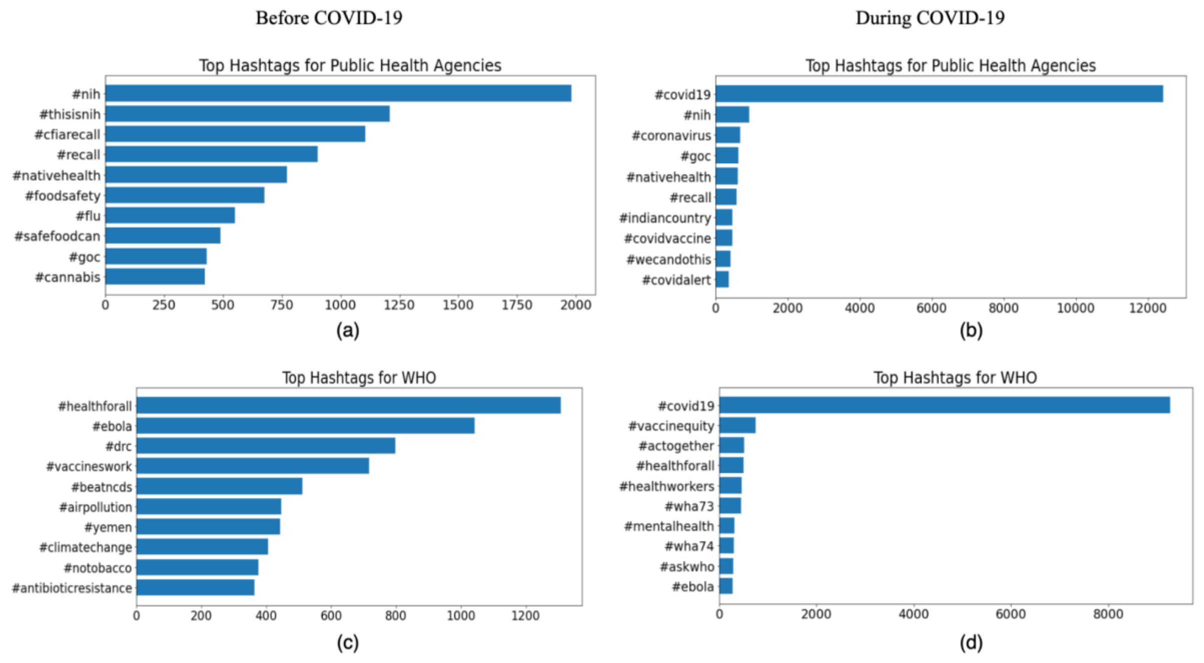

Figure S2: Top hashtags for different organizations *before COVID-19* and *during COVID-19 for Public Health Agencies and WHO*.

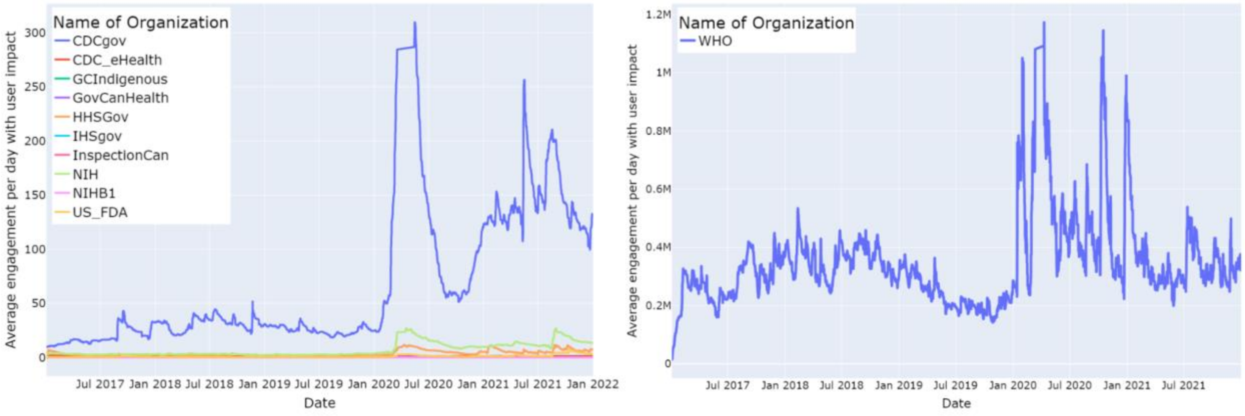

(a) Public Health Agencies (b) WHO

Figure S3: User Engagement on Twitter accounts of Public Health Agencies and WHO from January 01, 2017 to December 31, 2021

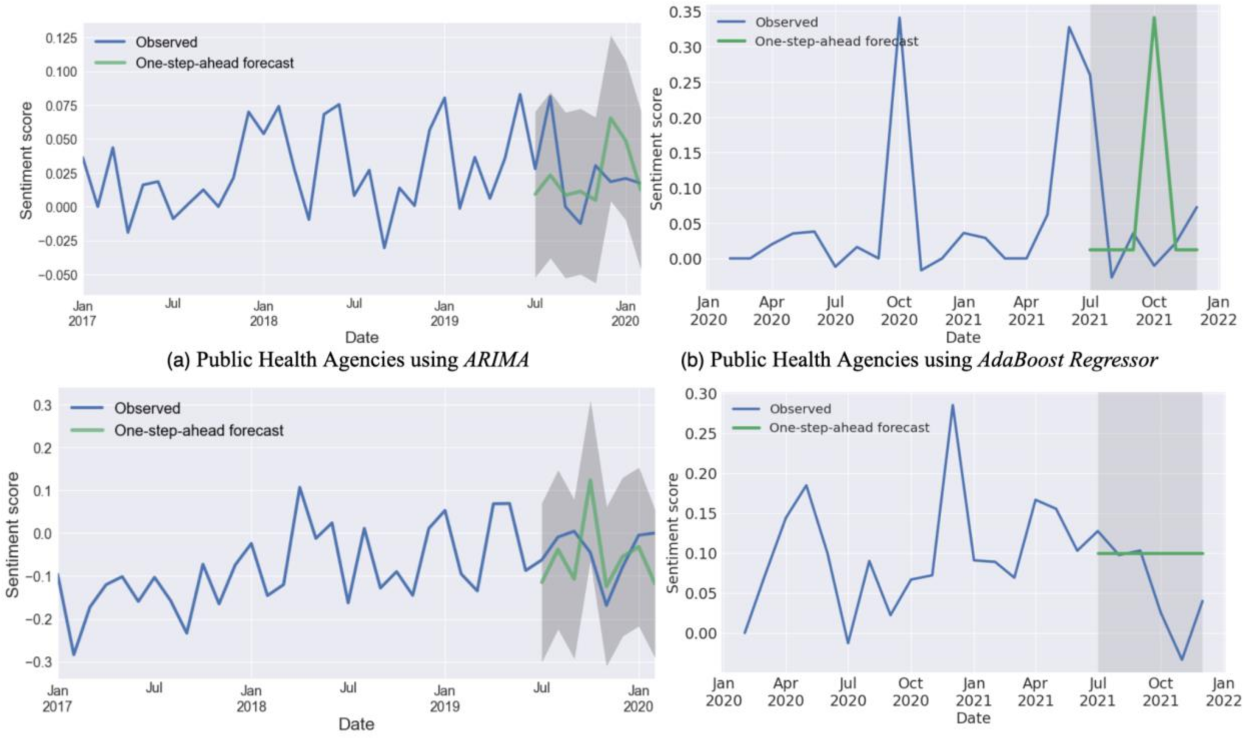

(a) Public Health Agencies using *ARIMA* (b) Public Health Agencies using *AdaBoost Regressor*

(c) WHO using *SARIMAX* (d) WHO using *Elastic Net*

Figure S4: One-step ahead forecast for Public Health Agencies and WHO *before COVID-19* and *during COVID-19* using the best performing models from Table S4.

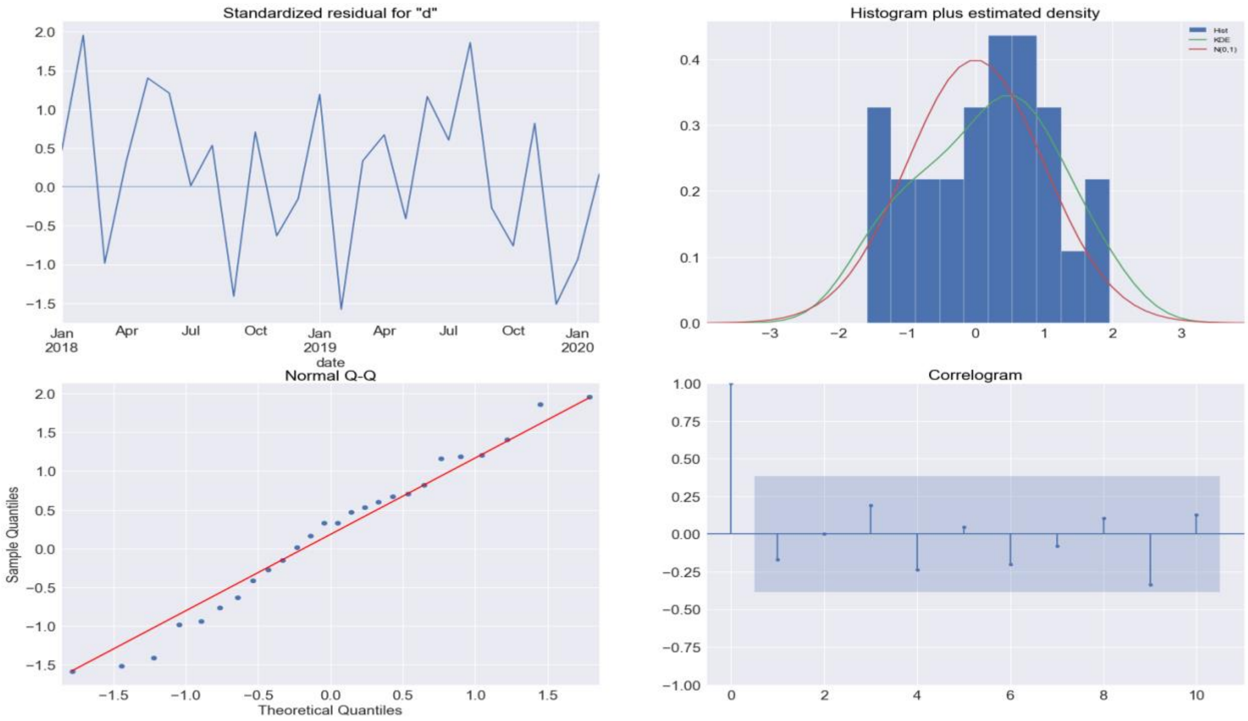

Figure S5: *plot_diagnostics* for Public Health Agencies *before COVID-19* using ARIMA.

The supplementary material for this study – data, code, and results are available on the GitHub repository (link).
